# Redlining−associated methylation in breast tumors: the impact of contemporary structural racism on the tumor epigenome

**DOI:** 10.3389/fonc.2023.1154554

**Published:** 2023-08-09

**Authors:** Jasmine M. Miller-Kleinhenz, Leah Moubadder, Kirsten M. Beyer, Yuhong Zhou, Anne H. Gaglioti, Lindsay J. Collin, Jazib Gohar, Whitney Do, Karen Conneely, Uma Krishnamurti, Keerthi Gogineni, Sheryl Gabram-Mendola, Olivia D’Angelo, Kashari Henry, Mylin Torres, Lauren E. McCullough

**Affiliations:** ^1^ Department of Epidemiology, Emory University Rollins School of Public Health, Atlanta, GA, United States; ^2^ Division of Epidemiology, Institute for Health & Society, Medical College of Wisconsin, Milwaukee, WI, United States; ^3^ National Center for Primary Care, Department of Family Medicine, Morehouse School of Medicine, Atlanta, GA, United States; ^4^ Center for Health Integration, Population Health Research Institute at The MetroHealth System, Case Western Reserve University, Cleveland, OH, United States; ^5^ Department of Population Health Sciences, Huntsman Cancer Institute, University of Utah, Salt Lake City, UT, United States; ^6^ Department of Global Health, Emory University Rollins School of Public Health, Atlanta, GA, United States; ^7^ Nutrition and Health Sciences Program, Laney Graduate School, Atlanta, GA, United States; ^8^ Department of Human Genetics, Emory University School of Medicine, Atlanta, GA, United States; ^9^ Department of Pathology and Laboratory Medicine, Emory University School of Medicine, Atlanta, GA, United States; ^10^ Department of Medical Oncology, Emory University School of Medicine, Atlanta, GA, United States; ^11^ Georgia Center for Oncology Research and Education, Atlanta, GA, United States; ^12^ Department of Surgery, Jackson Memorial Hospital/University of Miami Miller School of Medicine, Miami, FL, United States; ^13^ Department of Radiation Oncology, Emory University School of Medicine, Atlanta, GA, United States

**Keywords:** structural racism, breast cancer, health disparities, DNA methylation, epigenome, epigenetic age acceleration

## Abstract

**Purpose:**

Place-based measures of structural racism have been associated with breast cancer mortality, which may be driven, in part, by epigenetic perturbations. We examined the association between contemporary redlining, a measure of structural racism at the neighborhood level, and DNA methylation in breast tumor tissue.

**Methods:**

We identified 80 Black and White women diagnosed and treated for a first-primary breast cancer at Emory University Hospitals (2008–2017). Contemporary redlining was derived for census tracts using the Home Mortgage Disclosure Act database. Linear regression models were used to calculate the association between contemporary redlining and methylation in breast tumor tissue. We also examined epigenetic age acceleration for two different metrics, regressing β values for each cytosine-phosphate-guanine dinucleotide (CpG) site on redlining while adjusting for covariates. We employed multivariable Cox-proportional hazards models and 95% confidence intervals (CI) to estimate the association between aberrant methylation and mortality.

**Results:**

Contemporary redlining was associated with 5 CpG sites after adjustment for multiple comparisons (FDR<0.10). All genes were implicated in breast carcinogenesis, including genes related to inflammation, immune function and stress response (*ANGPT1, PRG4 and PRG4)*. Further exploration of the top 25 CpG sites, identified interaction of 2 sites (*MRPS28* and cg11092048) by ER status and 1 site (*GDP1*) was associated with all-cause mortality. Contemporary redlining was associated with epigenetic age acceleration by the Hannum metric (β=5.35; CI 95%=0.30,10.4) and showed positive but non-significant correlation with the other clock.

**Conclusion:**

We identified novel associations between neighborhood contemporary redlining and the breast tumor DNA methylome, suggesting that racist policies leading to inequitable social and environmental exposures, may impact the breast tumor epigenome. Additional research on the potential implications for prognosis is needed.

## Introduction

Breast cancer (BC) is one of the leading causes of cancer-related death among women in the United States (1). Despite having a similar incidence rate of BC to White women, Black women have higher rates of early-onset breast cancer (<40 years), greater incidence of more aggressive BC subtypes, and disproportionately die from their disease (2). Racial disparities in BC mortality are well documented, but the factors contributing to this disparity are not completely understood (3). Possible mechanisms for these differences have been investigated by examining racial differences in tumor biology (4). Black women are more likely to be diagnosed with the triple-negative breast cancer (TNBC) subtype, which, compared with other subtypes of BC, is usually detected at a later stage, has an aggressive pathology, and shorter overall patient survival. In fact, race disparities in BC mortality persist across BC subtypes (5). New approaches are necessary to further investigate racial disparities considering factors in addition to tumor biology.

Racial health inequities are often rooted in policies and structures that disproportionately affect minorities, especially Black Americans. Structural racism is the varied ways in which societies institute racial discrimination through systems such as housing, education, employment, earnings, benefits, credit, healthcare, and criminal justice (6). One example of a structural practice is redlining, defined as the systematic denial of mortgage loans based on location, most often occurs in locations defined by a particular race or socioeconomic group (7). Redlining has precipitated the disinvestment of certain geographic areas, influencing a multitude of determinants of health, including housing, education, physical work environment, healthcare, nutrition, greenspace, physical activity, and financial stress (8–12). Redlining and other discriminatory housing practices resulting in segregation have been operationalized as a measure of structural racism (13–16). Redlining, as an exposure, may capture a combination of adverse social and environmental factors (structural racism) beyond just measuring neighborhood characteristics or socioeconomic status (SES) alone. It may also serve as a proxy for exposure to chronic stressors or economic disinvestment. Recent work has identified both historic and contemporary measures of redlining and racial bias in mortgage lending as measures of structural financial inequities linked with poorer BC survival (7, 17).

There is little information concerning how structural determinants of health impact BC outcomes. Recent studies have linked neighborhood characteristics such as neighborhood deprivation (18), job density and college graduation rates (19) with differential cytosine-phosphate-guanine (CpG) methylation in breast tumor tissue. Many of these CpGs are within genes that have been implicated in carcinogenesis and provide potential insight into the biological impact of neighborhood stressors on the breast tumor epigenome. Additionally, given that cancer is a disease characterized by the process of aging, measures of epigenetic age acceleration may reflect adverse neighborhood exposures and BC prognosis, in addition to chronologic age. Thus, examining epigenetic perturbations can help elucidate the relationship between exposure to structural-socio factors and biological alterations within the tumor which may contribute to outcome disparities (20).

Elucidating the biological mechanisms underlying the associations between systemically racist policies and BC prognosis is necessary to conceive appropriately targeted multi-level interventions. To date, no study has used a genome-wide approach to assess redlining-associated methylation signatures and outcomes in tumor tissue of women diagnosed with BC. The goal of this pilot investigation was to conduct an epigenome-wide association study (EWAS) of breast tumor tissue to identify novel differentially methylated CpG sites associated with contemporary redlining. We additionally assessed the association between contemporary redlining and epigenetic age acceleration using different epigenetic clocks.

## Methods

### Study population

Our study received IRB approval from Emory University (IRB00018512). All specimens were procured from Winship’s Breast Cancer Satellite Bank, which receives written informed consent from patients for tissue collection. Study protocol follows the methodology described in Do et al. (21). Briefly, fresh tumor specimens and clinical data were collected from patients undergoing surgery at three metro-Atlanta area hospitals (Emory University Hospital, Emory University Hospital Midtown, and Grady Memorial Hospital). We included 17 non-Hispanic White (NHW) and 63 non-Hispanic Black (NHB) women diagnosed with BC between 2008 and 2017 in this analysis. Eligibility for inclusion were: women who were at least 21 years of age; self-reported NHW or NHB race/ethnicity; diagnosed with a first-primary stage I, II, or III BC, and underwent surgery at one of the above institutions. Additionally, women were included if they resided in the metro-Atlanta area at the time of diagnosis, which includes Cobb, Clayton, DeKalb, Fulton, and Gwinnett counties, and if their reported address was within a census tract that could be geocoded. Women were excluded from this study if they were previously diagnosed with BC or did not have a fresh tissue specimen available.

### Exposure assessment

We define redlining as a systematic denial of mortgage based on location. Contemporary redlining was calculated based on a previously published methodologic approach by Beyer et al. (17). Briefly, data were abstracted from the national database established as part of the Housing Mortgage Disclosure Act (HMDA) for the years 2010–2014 (22, 23). The database collects information on mortgage lending practices, including location for which a mortgage was being requested (census tract); loan approval/denial; loan type (purchase/refinance) and amount; owner-occupancy; and the applicant’s race/ethnicity, sex, and income.

The redlining index was calculated as described in Collin et al. 2020 and Beyer et al. 2021 (24, 25). Briefly, the index was estimated across a set of estimation points using adaptive spatial filters, as the odds of denial of a mortgage application for a residence inside the spatial filter as compared to properties in the rest of the metropolitan statistical area. The index centers around a value of one, where >1 means that applicants applying for mortgages for properties in that neighborhood are more likely to be denied mortgage applications than applicants applying for mortgages for properties in other areas and a value <1 means that the applicants for properties in that neighborhood are less likely to be denied. The index is averaged at the census tract level. Using the patient’s address at diagnosis, we assigned the area level measure for redlining to the patients residing in those census tracts. The redlining score was examined by quartiles (Q1 ≤1, Q2 < 2.5, Q3< 4, Q4<7.7) for the genome wide DNA methylation analyses and dichotomized at <1 for the epigenetic age analyses.

### Outcome assessment

Updated vital status (through 2/15/2018) was obtained by linking all women to the Georgia Cancer Registry, and all-cause and cause-specific death was abstracted. As described in Do et al., we considered all-cause mortality as an outcome of interest (21). Given the short follow-up period (median = 3 years) we expect any mortality event would be, in part, driven by the underlying BC (26). We additionally conducted a sensitivity analysis to examine the association with breast cancer-specific mortality.

### Covariates of interest

Patient characteristics and covariate data were provided from the clinical records of women who underwent surgery. Covariates included age at diagnosis, race/ethnicity, body mass index (BMI, kg/m^2^), insurance status, educational attainment, family history of BC, and self-reported smoking status. Tumor characteristics were also obtained from clinical records including estrogen receptor (ER) status, tumor stage; receipt of chemo-, radiation, or endocrine therapy; and comorbidities at diagnosis.

### DNA methylation data

We acquired fresh breast tumor specimens from the Glenn Family Breast Satellite Tissue Bank, Winship Cancer Institute, Emory University, Atlanta, GA, USA. All specimens were reviewed visually by a breast pathologist (UK) to enrich for tumor cells. We then utilized the Emory Integrated Genomics Core for DNA extraction which extracts genomic DNA from tissue using the QIAamp DNA Mini Kit (Qiagen; 51306). DNA methylation was measured in 80 breast tumor tissue samples using the Illumina Infinium MethylationEPIC 850K Beadchip (Illumina, San Diego, CA, USA). Methylation assays were performed in accordance with the Infinium HD Methylation Assay protocol. The DNA methylation values represent the portion of methylated sites to the sum of total methylated (M) and unmethylated (U) sites at given CpG site (27). The generated β-value is calculated as β =[M/(M+U)]. β-values range from 0 to 1, where 1 represents 100% of the probes being methylated at a CpG site.

Quality control (QC) was conducted on the data using the *CpGassoc* package in R (28). Data points with low signal or detection p-values >0.001 were set to missing, and CpG sites with missing values (2,869) in >10% of the samples were removed from the dataset. As suggested by Zhou et al. stricter probe filtering was utilized by filtering out CpG sites which include; (1) probes with low quality or inconsistent mapping, (2) probes with non-unique sub-sequences, (3) probes with non-unique hybridization (4) probes with single nucleotide polymorphisms (SNP) at a minor allele frequency >1%, and (5) probes with a SNP that causes a color channel switch (29). After QC, 758,942 CpG sites remained for evaluation with our redlining metrics.

### Calculation of epigenetic age

Epigenetic age was based on two different clocks—Horvath et al. (30), and Hannum et al. (31), and was calculated using the *methylclock* R package (32). Briefly, the package extracts methylation levels from CpGs used in each clock. The coefficients from the original papers, which were obtained via prediction models, were used to calculate DNA methylation age and epigenetic age acceleration for our study population. For each clock, we obtained: 1) DNA methylation predicted age (DNAm age) in years, and 2) age acceleration (ageAcc), the difference between DNAm and chronological age in years. Positive values for epigenetic age acceleration indicated that epigenetic age is higher than chronological age.

### Statistical analysis

Patient demographic and clinicopathologic characteristics were reported overall and by race/ethnicity as means and corresponding standard deviations.

Linear regression models were used to assess whether individual mean β-values differed by contemporary redlining, adjusting for model-specific covariates based on *a priori* knowledge of the literature and causal diagrams (33, 34). Using the CpGassoc R package, we regressed β-values for each CpG site on redlining, adjusting for age, race/ethnicity, smoking status, and chip position (28). Models also included a fixed effect for each BeadChip to account for potential chip-to-chip differences in measurement and to adjust for potential batch effects. For epigenome-wide analyses, statistical significance was defined as a false discovery rate (FDR) of q-value < 0.10. We similarly used linear regression to estimate the association between redlining and epigenetic age acceleration in unadjusted models and models adjusted for age and race/ethnicity.

To assess whether the association between redlining and tumor methylation was modified by ER status, the CpG sites reaching FDR significance in the primary EWAS were tested for interaction. For each CpG site, β-values were regressed on redlining with an interaction between the redlining and ER status. All interaction analyses were adjusted for age and chip position, with significance defined as FDR < 0.10.

We used multivariable-adjusted Cox proportional-hazards models to explore associations between the top redlining-associated CpG sites and all-cause mortality. We computed hazard ratios (HRs) and corresponding 95% confidence intervals (CIs) adjusting for (1) age, (2) age and race/ethnicity, and (3) age, race/ethnicity, cancer stage, and ER status. Additionally, we evaluated the association between age acceleration and all-cause mortality. We calculated HRs and 95% CIs for crude, age, and race/ethnicity-adjusted models. All analyses were carried out using R v4.1.0 and R v4.3.0 (Vienna, Austria).

## Results

Demographic data for our study population are provided in **Table 1**. NHB women were, on average, older (mean age = 57.6 and 51.3 years, respectively) compared with NHW women and had a higher BMI (mean BMI = 38.7 vs 30.9 kg/m^2^, respectively). NHB women, compared with NHW women, more often resided in redlined neighborhoods (defined as ≥1, 88.9% vs. 29.4%, respectively) and lived neighborhoods with a greater redline index (mean index= 2.59 and 0.65, respectively) (**Supplemental Figure 1**). No difference between NHB and NHW women were observed by ER status, family history of BC, and all-cause mortality.

**Table 1:** Patient demographic and clinicopathologic characteristics among 80 NHB and NHW women diagnosed with stage I to III breast cancer in metropolitan Atlanta between 2010–2014.

	Total		NHB (n=63)		NHW (n=17)	
**Age at diagnosis Mean** (SD)	55.1	13.5	57.6	13.2	51.3	11.5
Age						
≤49	23	28.7	15	23.8	8	47.1
50-59	29	36.2	23	36.5	6	35.3
≥60	28	35.0	25	39.7	3	17.6
Redlining						
0	19	23.7	7	11.1	12	70.6
1	61	76.2	56	88.9	5	29.4
**Redlining Index Mean** (SD)	2.17	1.98	2.59	1.96	0.65	0.47
Stage						
I	26	32.5	20	31.8	6	29.4
II	42	52.5	34	54.0	8	47.06
III	8	10.0	7	11.0	1	5.9
Unknown	4	5.0	2	3.2	2	11.8
ER Status						
Positive	62	77.5	50	79.4	12	70.6
Negative	18	22.6	13	20.6	5	29.4
**BMI** Mean (SD)	37.1	30.2	38.7	33.7	30.9	6.32
Insurance						
Private	7	8.8	3	4.8	4	23.5
Medicaid	32	40.0	27	42.9	5	29.4
Medicare	29	36.3	27	42.9	2	11.8
Uninsured	9	2.5	3	4.8	6	35.3
Unknown	3	3.8	3	4.8	2	11.8
Education						
Some high school	6	7.5	4	6.3	2	11.8
Highschool diploma/GED	38	47.5	34	54.0	4	23.5
Some college	15	18.8	12	19.0	3	17.6
Bachelors or higher	14	17.5	7	11.1	7	41.2
Unknown	7	8.8	6	9.5	1	5.9
Family history						
Yes	17	21.3	14	22.2	3	17.6
No	63	78.7	49	77.8	14	82.4
Tobacco history						
Yes	32	40	29	46	3	17.6
No	47	58.8	33	52.4	14	82.4
Unknown	1	1.2	1	1.6	0	–
Chemotherapy						
Yes	44	55	34	54	10	59
No	36	45	29	46	7	41
Endocrine Therapy						
Yes	55	69	42	67	13	76
No	22	28	18	29	4	24
Unknown	3	3	3	4		
Radiation Therapy						
Yes	40	50	32	51	8	47
No	33	41	26	41	7	41
Unknown	7	9	5	8	2	12
All-Cause Mortality						
Yes	21	26.3	17	27.0	4	23.5
No	53	73.8	46	73.0	13	76.5

**Figure 1:**
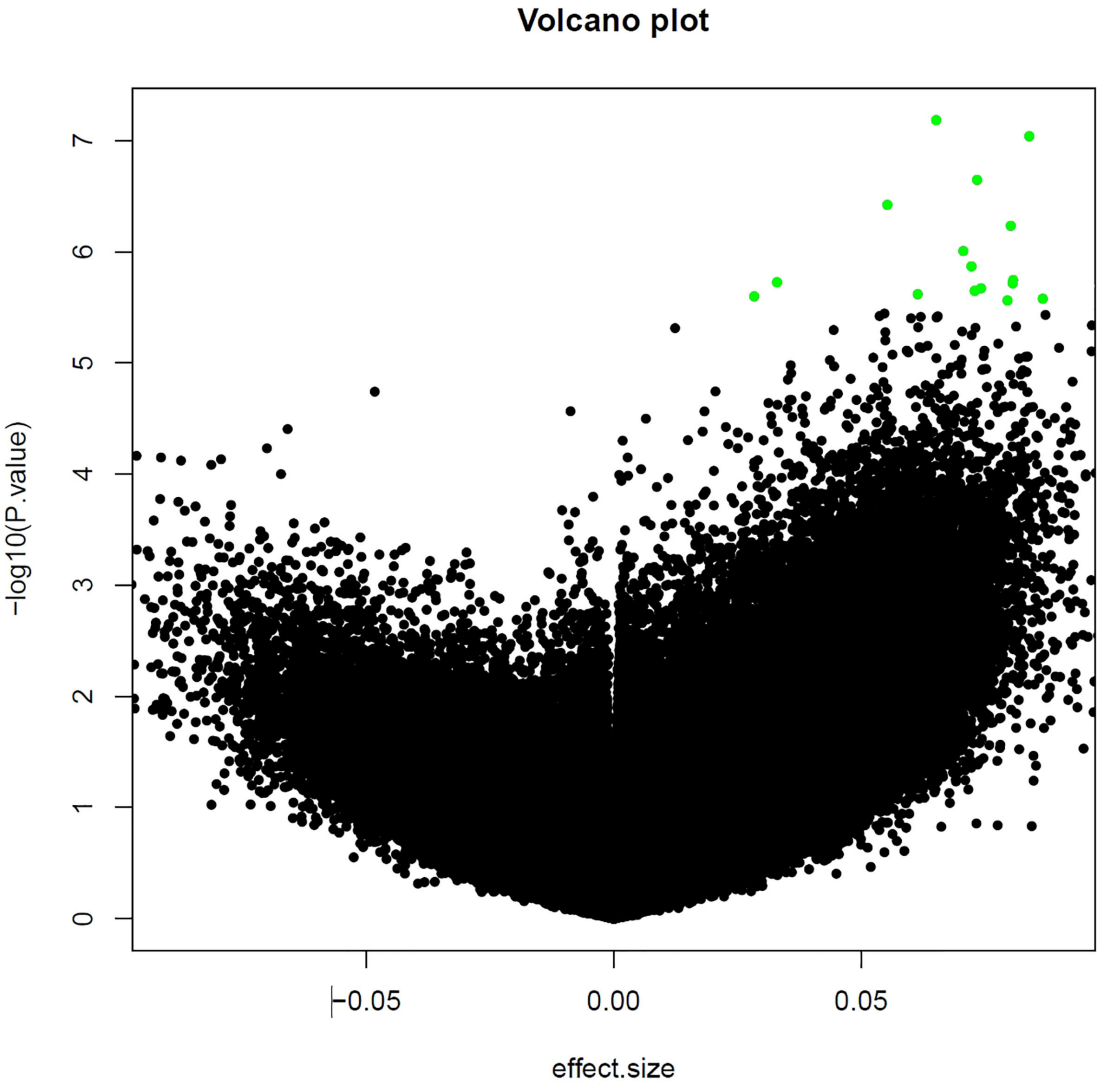
Volcano plot of CpG sites associated with neighborhood-level redlining. The top 25 CpG sites are those shown in green.

For the epigenome wide analysis assessing the association of DNA methylation with contemporary redlining, we found 5 CpG sites that passed our threshold of FDR significance after adjustment for multiple comparisons (FDR<0.10), with two of the CpG sites having an FDR<0.05 (**Table 2**). All 5 CpGs were hypermethylated (**Figure 1**). Contemporary redlining was also positively correlated with increased age acceleration using both clocks, though only the Hannum measure reached conventional significance levels (*P*<0.05) (**Table 3**). Of the first-generation clocks, the increase in age acceleration associated with redlining was higher with Hannum (β = 5.35; 95% CI=0.30,10.4) compared to Horvath (β = 4.04; 95% CI=-1.72,9.82) in age-adjusted models. After adjusting for race, we saw a more pronounced association with Hannum (β = 6.61; 95% CI=0.5,12.7) while the association for Horvath was attenuated upon adjustment for race (β = 2.81; 95% CI= -4.15,9.78).

**Table 2:** The top 25 FDR-significant CpG sites associated with a neighborhood-level redlining in breast tumor tissue in EWAS.

CpG Label	T-statistic	p-value	FDR	Effect Size	Std Error	Gene Name	Gene Region	Chr
cg06081220	6.241	6.55E-08	0.035	0.065	0.010	PCDH9	Body	13
cg23248351	6.151	9.15E-08	0.035	0.084	0.014	ANGPT1	TSS1500	8
cg20275129	5.908	2.26E-07	0.057	0.073	0.012			13
cg13274183	5.769	3.79E-07	0.072	0.055	0.010	PRG4	TSS1500	1
cg27569887	5.651	5.85E-07	0.089	0.080	0.014	RBMS3	3’UTR	3
cg00059737	5.343	1.88E-06	0.110	0.033	0.006	VPS13D	Body	1
cg01020413	5.500	1.02E-06	0.110	0.106	0.019			2
cg01495275	5.274	2.41E-06	0.110	0.061	0.012	CUL3	Body	2
cg02267536	5.240	2.62E-06	0.110	0.110	0.021	BANP	Body	16
cg04922153	5.509	9.86E-07	0.110	0.071	0.013			20
cg06649682	5.422	1.35E-06	0.110	0.072	0.013	MRPS28	Body	8
cg11053632	5.309	2.04E-06	0.110	0.098	0.018	BANP	Body	16
cg11092048	5.237	2.65E-06	0.110	0.087	0.017			15
cg11675630	5.325	1.92E-06	0.110	0.081	0.015	FAM179A	Body	2
cg14402950	5.308	2.13E-06	0.110	0.074	0.014	LOC101927967	Body	2
cg15073453	5.227	2.75E-06	0.110	0.080	0.015	KIRREL3	Body	11
cg15239796	5.250	2.52E-06	0.110	0.028	0.005	EXOG	Body	3
cg16747973	5.282	2.25E-06	0.110	0.073	0.014	FGG	5’UTR	4
cg27611830	5.343	1.80E-06	0.110	0.081	0.015	PKNOX2	Body	11
cg00257769	5.152	3.60E-06	0.112	0.055	0.011	PARP4	Body	13
cg08808042	5.128	3.92E-06	0.112	0.101	0.020	BANP	Body	16
cg09589360	5.132	3.86E-06	0.112	0.062	0.012	OVCH1	TSS1500	12
cg11683511	5.137	3.79E-06	0.112	0.054	0.010	GPD1	TSS1500	12
cg12869679	5.144	3.70E-06	0.112	0.087	0.017			8
cg13161621	5.136	3.81E-06	0.112	0.065	0.013	IQSEC2	Body	X

T-statistics, p-value and false discovery rate q-value, effect size, and standard error have been provided. EWAS was adjusted for age, race, smoking status, and batch effects. Reference gene name, gene region, and chromosome number obtained from the Illumina annotation file. Blank spaces represent intergenic regions.

**Table 3:** Association between neighborhood-level redlining and epigenetic acceleration (1) age, and (2) age and race.

Clock	Horvath		Hannum	
Age Acceleration	HR (95% CI)	p-value	HR (95% CI)	p-value
Age-adjusted	4.04 (−1.72,9.82)	0.167	5.35(0.30,10.4)	0.038
Age and Race-adjusted	2.81(−4.15,9.78)	0.424	6.61(0.5,12.7)	0.034

Due to the exploratory nature of the analysis, we performed interaction analyses to examine the differences in redlining and DNA methylation in the top 25associated CpG sites by ER status (**Table 2**). There were two CpG sites, cg06649682 (*MRPS28*) and cg11092048 (non-coding RNA), where the relationship between redlining and DNA methylation differed by ER status after adjustment for multiple comparisons (FDR <0.1). While we observed a positive association between redlining and methylation across both CpG sites, women with ER-negative BC had significantly higher methylation than ER-positive BC (**Supplemental Figures 2A, B**).

Of the top 25CpG sites associated with redlining, we found 1 CpG, cg11683511(*GPD1*), associated with mortality in a multivariable model accounting for age, race, clinical stage, and ER status with a 17% increase risk observed with every 1% increase in methylation (HR= 1.17 95%CI 1.02,1.35), although these results did not pass the threshold for FDR significance. (**Supplemental Table 1**). We additionally explored whether epigenetic age contributed to all-cause mortality and found the Horvath clock was associated with a slight reduction in hazard of death in multivariable models (**Table 4**). In a sensitivity analysis including breast cancer-specific mortality we observed that the Horvath clock was similarly associated with a slight reduction in a hazard of death in multivariable models (**Supplemental Table 2**).

**Table 4:** Hazard ratio and 95% CI associating epigenetic aging with all-cause mortality (1) unadjusted, (2) age, and (3) age, and race.

Deaths (*n=21)*	Horvath		Hannum	
Age Acceleration	HR (95% CI)	p-value	HR (95% CI)	p-value
Unadjusted	0.96 (0.93, 0.99)	0.007	0.97 (0.93,1.00)	0.06
Age-adjusted	0.96 (0.93, 1.00)	0.07	0.98 (0.93,1.03)	0.4
Age and Race-adjusted	0.96 (0.93,1.00)	0.07	0.97 (0.93, 1.03)	0.35

## Discussion

Despite accumulating evidence supporting the hypothesis that SES may be associated with increased cancer risk and poor cancer outcomes through epigenetic mechanisms, limited studies have reported the association between adverse sociodemographic characteristics, breast tumor methylation, and mortality (35). This pilot study is the first untargeted analysis to examine redlining-associated methylation in breast tumor tissue. We identified 5 CpG sites associated with contemporary redlining. We also report that contemporary redlining was associated with epigenetic age-acceleration using two different epigenetic clocks. Given that Black women, compared to their White counterparts, were disproportionately exposed to contemporary redlining (88.9% vs. 29.4%, respectively), structural racism may differentially impact Black women with BC in the metropolitan-Atlanta area.

Most of the 5 CpG sites associated with redlining were in the transcription start site or body of genes that have been characterized as tumor suppressors and implicated in carcinogenesis. Similar to other studies that have seen racial discrimination manifest biologically through activation of stress pathways (36), we found the differentially methylated CpGs within genes involved in inflammation, immune function, and response to stress through gene ontology analysis including *ANGPT1*, *PRG4*, and *RBMS3*. Angiopoietin-1 (*ANGPT1)* is an important regulator of inflammation and angiogenesis, and stimulates monocytes through ERK 1/2 phosphorylation (37). Research has shown that inhibition of ANGPT1 and its receptor, Tie2, results in human breast cancer cells becoming more sensitive to antigen-specific cytotoxic lymphocytes (38). Proteoglycan 4 (PRG4), is involved in diverse biological processes including anti-inflammation, cytoprotection, and anti-adhesion, amongst others (39). PRG4 is also involved in the regulation of transforming growth factor beta (TGF-β) (40). PRG4 suppresses TGFβ-induced invasiveness of breast cancer cells by inhibiting the cell surface cluster of differentiation 44 (CD44) signaling (40). RNA Binding Motif Single Stranded Interacting Protein 3 (RBMS3) is a known tumor suppressor in breast cancer and is significantly associated with the epithelial to mesenchymal (EMT) transition, especially in TNBC (41, 42). RBMS3, through inactivation of the Wnt/β-catenin signaling pathway, also inhibits proliferation and tumorigenesis of breast cancer cells (43). In addition, RBMS3 is a crucial regulator of programmed death ligand-1 (PD-L1) in TNBC and through post-transcriptional regulation of PD-L1, RMBS3 can contribute to immune escape in TNBC (44). In addition, *ANGPT1*, *PRG4*, and *RBMS3* were found to be mutated within a portion of the TCGA breast cancer samples. Given the posited role of these genes, our collective data support a potential biological mechanism linking neighborhood-level structural racism and the regulation of inflammatory and immune pathways.

We also found redlining was positively correlated with epigenetic age acceleration using two clocks, though only the Hannum clock surpassed conventional significance levels. The Horvath and Hannum clocks are commonly used first-generation clocks related to chronological age alone (30, 31, 45). While the Hannum clock was trained in whole blood a recent study showed that the CpGs comprising the clock are able to strongly and consistently predict age correlations across several tissue, including breast tumor tissue, and cell types (46). Our findings support recent research showing that neighborhood deprivation accelerates epigenetic age for NHW women, which was characterized using multiple epigenetic clocks, including Hannum (18). In addition, our data align with previous studies among Black women that showed higher neighborhood deprivation associated with higher epigenetic age acceleration when measured by the Hannum clock (47). The early age onset of aggressive BC in Black women parallels a growing body of scientific evidence that demonstrates the earlier age onset of chronic health conditions in Black compared with White persons (48). This emphasizes the need to investigate how racial differences in social-environmental exposures impact disparities in disease risk, morbidity, and mortality. The sustained and cumulative exposure to adverse social, physical, psychological, and chemical stressors within their residential, educational, employment, and other environments, may result in biological weathering among disadvantaged populations (49). Biological weathering, secondary to experiences of structural racism, could be related to the observed epigenetic age acceleration and may explain the earlier onset and poorer prognosis of Black persons with chronic illnesses, including BC.

Of the top 25 CpG sites identified as being differentially methylated by exposure to redlining, modification by ER status was found in two sites, cg06649682 (*MRPS28*) and cg11092048 (non-coding RNA). Mitochondrial Ribosomal Protein S28 (MRPS28) is a member of a family of proteins which are involved with mitochondrial energy metabolism which have been implicated in breast cancer prognosis and members of the MRP family have been shown to differentially expressed based on breast cancer subtype (50). For both CpG sites, ER-negative tumors had greater redlining-associated methylation than ER-positive tumors, suggesting there are potential implications of racist housing polices on tumor aggressiveness.

One site, cg11683511(GPD1), was associated with contemporary redlining and all-cause mortality. Glycerol-3-phosphate dehydrogenase 1 (GPD1) is a tumor suppressor within breast cancer and reduced expression of GDP1 is associated with poor overall survival (51). We found that this site was hypermethylated within the transcription start site, which is indicative of decreased expression. Given the suppressive function of GDP1, our results further support the role of GDP1 in breast cancer prognosis. We observed only slight associations between epigenetic age acceleration and all-cause mortality, with the Horvath clock being associated with a slight reduction in hazard of mortality. While these results were unanticipated, our overall epigenetic age acceleration findings could indicate that the role of epigenetic age in the etiology of breast cancer differs from its role in mortality. Future studies will require a larger sample size and more complete follow-up to further evaluate the effect of redlining-associated DNA methylation on prognosis.

Our study has several limitations. First, we only included 80 NHB and NHW women from a single hospital system in this study, however we were still able to identify redlining-associated differential methylation at5 CpG sites even after adjusting for multiple comparisons. The timeframe for our study population (2008-2017) and redlining metric (2010-2014) do not completely overlap which could lead to potential exposure misclassification. In addition, we only have patients address at exposure so we are unable to assess residential history or patient mobility and therefore the duration of their exposure to living in a redlined area. There are limited data on neighborhood mobility among NHB people, but emerging evidence suggests that they often move to areas that have similar characteristics to their previous residence (52–54). While we do not have residential history, it is likely that our residents lived in redlined areas for a sustained period of time. We did not have sufficient sample size to examine associations of DNA methylation on all-cause mortality by ER status. We also used tumor tissue to measure associations between epigenetic age and outcome. Epigenetic age in normal and tumor tissue might manifest differently, using either tissue might not be representative of the other and our epigenetic age results focus exclusively on the impact of tumor tissue pathology outcome. More robust studies will be needed to substantiate and expand our findings. Particularly, it will be important for future studies to explore the functional significance of the observed perturbations. Finally, we exclusively focus on the DNA methylome, which negates other layers of the epigenome and downstream impacts on gene expression that may also play a role in BC outcomes.

This study employed both discovery-based and CpG site-specific approaches to examine potential epigenetic mechanisms underlying the association between contemporary redlining and BC prognosis in a diverse population of women residing in metro-Atlanta. Our preliminary results suggest that neighborhood-level exposure to structural racism has biological consequences and is associated with unfavorable perturbations of the breast tumor DNA methylome. Understanding the structural factors that influence health outcomes at the biological level is necessary to identify appropriate interventions. Future studies should be conducted with a larger, equally diverse patient population to validate our preliminary findings and further interrogate the epigenetic perturbations identified in this study.

## Data availability statement

The datasets generated during and/or analyzed during the current study are not publicly available due to IRB protocol but are available from the corresponding author on reasonable request to study PI (lauren.mccullough@emory.edu).

## Ethics statement

The studies involving human participants were reviewed and approved by IRB approval from Emory University (IRB00018512). The patients/participants provided their written informed consent to participate in this study.

## Author contributions

JM-K: Conceptualization, data curation, formal analysis, methodology, writing–original draft, writing–review and editing. LEM: Conceptualization, data curation, methodology, writing–original draft, writing–review and editing. KB: Data curation, writing–review and editing. YZ: Data curation, writing–review and editing. AG: Conceptualization, methodology, writing–review and editing. LC: methodology, writing–review and editing. JG: Validation, methodology, writing–review and editing. WD: Validation, methodology, writing–review and editing. KC: Supervision, formal analysis, methodology, writing–review and editing. UK: data curation, writing–review and editing. KG: funding acquisition, writing–review and editing. SG-M: funding acquisition, writing–review and editing. OD’A: data curation, writing–review and editing. MT: data curation, writing–review and editing. KH: Formal analysis, visualization, writing–review and editing. LM: Conceptualization, resources, data curation, supervision, funding acquisition, methodology, writing–original draft, project administration, writing–review and editing. All authors contributed to the article and approved the submitted version.
