# Supplementary material for: Redlining−associated methylation in breast tumors: the impact of contemporary structural racism on the tumor epigenome
Source: Front Oncol. 2023 Aug 9;13:1154554. doi: 10.3389/fonc.2023.1154554 (PMC10446968; doi:10.3389/fonc.2023.1154554)
Supplement: Supplementary file 1 [file DataSheet_1.docx]

Supplementary Material

**Title:** Redlining‑associated methylation in breast tumors: the impact of contemporary structural racism on the tumor epigenome

**Author List:** Jasmine M Miller-Kleinhenz^*^, Leah Moubadder, Kirsten M Beyer, Yuhong Zhou, Anne H. Gaglioti, Lindsay J Collin, Jazib Gohar, Whitney Do, Karen Conneely, Uma Krishnamurti, Keerthi Gogineni, Sheryl Gabram-Mendola, Olivia D'Angelo, Kashari Henry, Mylin Torres, Lauren E. McCullough

**Contact Information**: Jasmine Miller-Kleinhenz, email: jmill37@emory.edu

**Supplemental Figure I.** Density plot of distribution of continuous redlining index by race/ethnicity, including non-Hispanic Black (NHB) and non-Hispanic White (NHW) women diagnosed with breast cancer in metro-Atlanta (2008-2017).

**Supplemental Figure II.** Scatter plot and regression line depicting *β*-values by contemporary redlining. **A-B** Examines the interaction by ER status for cg06649682 and cg11092048, respectively.


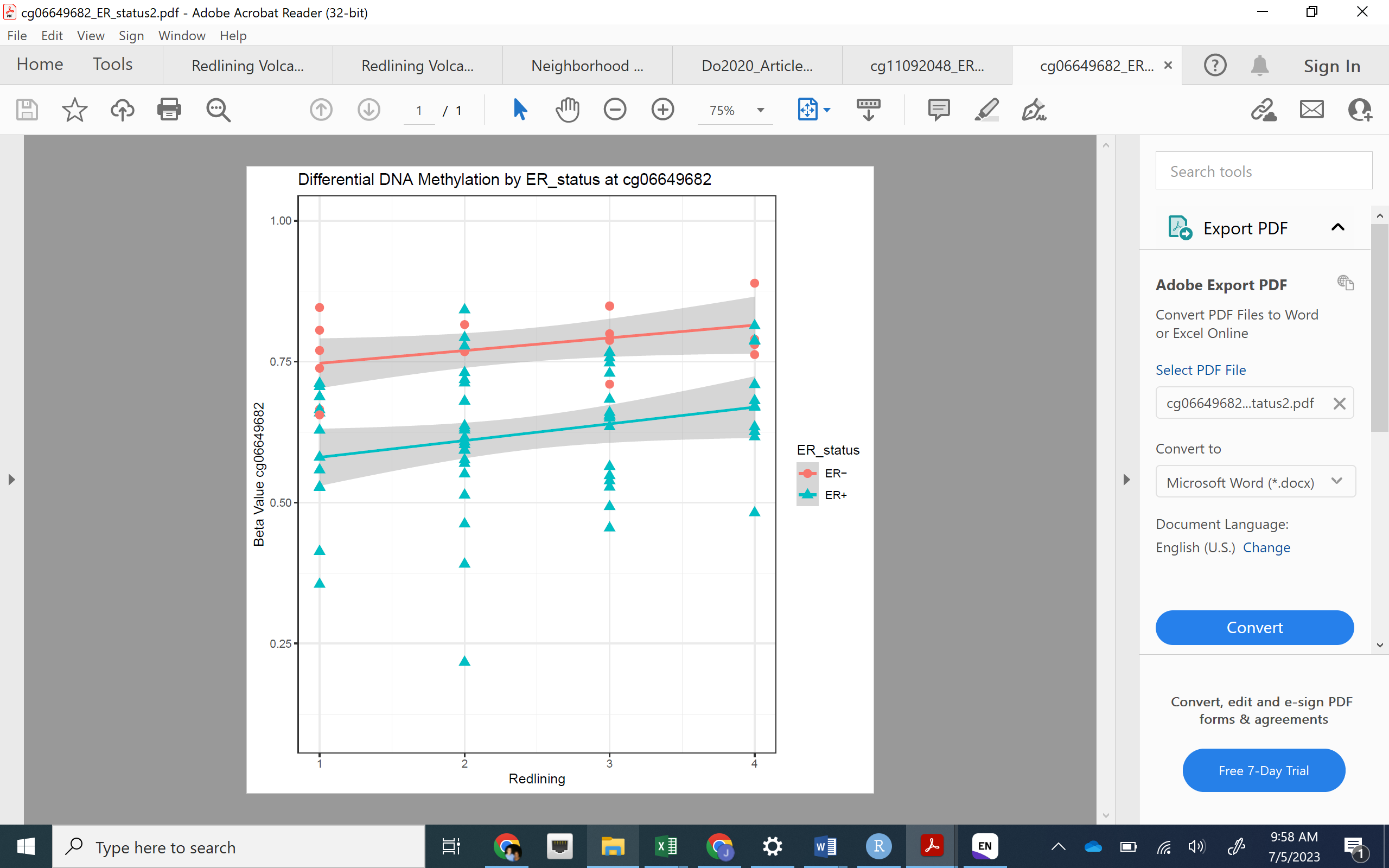

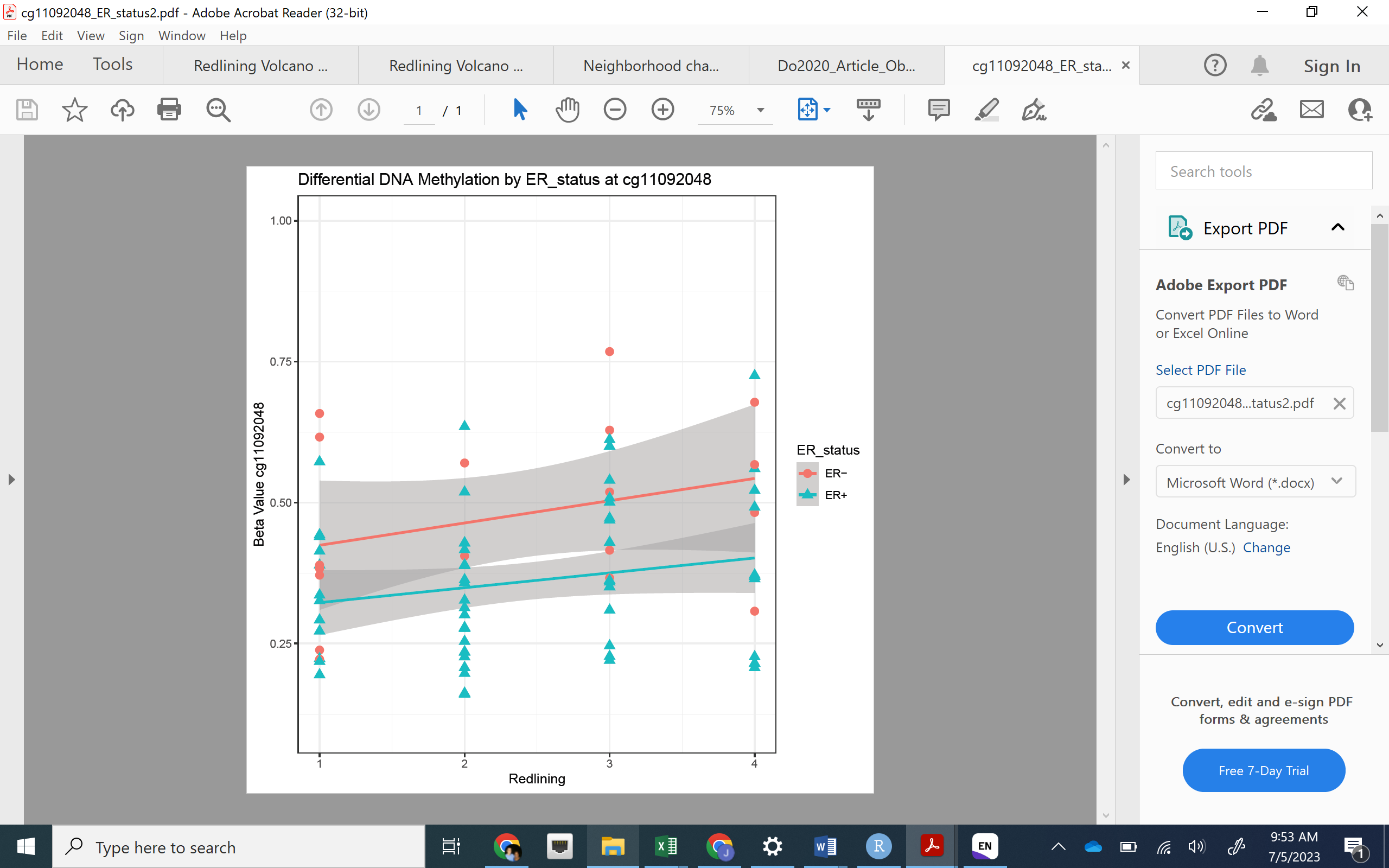


**Supplemental Table I.** Hazard ratios models associating the top 25 neighborhood-level redlining-associated CpG sites with all-cause mortality adjusted for age.

| **CpG Label** | **Adjusted for age HR (95% CI)** | **Adjusted for age and Race HR (95% CI)** | **Adjusted for age, Race, Stage and ER Status HR (95% CI)** |
| --- | --- | --- | --- |
| cg06081220 | 0.97 (0.92,1.01) | 0.97 (0.92,1.01) | 0.95 (0.90,1.01) |
| cg23248351 | 0.93 (0.97,1.03) | 1.00 (0.96,1.03) | 0.98 (0.94,1.03) |
| cg20275129 | 0.98 (0.97,1.04) | 1.00 (0.97,1.04) | 0.99 (0.95,1.04) |
| cg13274183 | 1.01 (0.96,1.06) | 1.01 (0.96,1.06) | 1.01 (0.95,1.07) |
| cg27569887 | 1.00 (0.97,1.04) | 1.00 (0.97,1.04) | 0.99 (0.94,1.04) |
| cg00059737 | 0.91 (0.81,1.03) | 0.92 (0.81,1.04) | 0.90 (0.76,1.05) |
| cg01020413 | 1.03 (0.99,1.06) | 1.02 (0.99,1.06) | 1.04 (0.99,1.09) |
| cg01495275 | 1.03 (0.97,1.09) | 1.02 (0.96,1.09) | 1.04 (0.96,1.12) |
| cg02267536 | 1.01 (0.97,1.04) | 1.00 (0.97,1.04) | 0.98 (0.93,1.04) |
| cg04922153 | 1.00 (0.96,1.04) | 1.00 (0.96,1.04) | 0.98 (0.93,1.04) |
| cg06649682 | 1.01 (0.97,1.05) | 1.01 (0.97,1.04) | 1.01 (0.96,1.07) |
| cg11053632 | 1.01 (0.97,1.05) | 1.01 (0.97,1.05) | 1.01 (0.95,1.08) |
| cg11092048 | 1.01 (0.97,1.06) | 1.01 (0.97,1.06) | 1.02 (0.96,1.08) |
| cg11675630 | 1.01 (0.97,1.06) | 1.01 (0.97,1.05) | 1.02 (0.96,1.08) |
| cg14402950 | 1.02 (0.97,1.08) | 1.03 (0.97,1.09) | 1.02 (0.96,1.09) |
| cg15073453 | 0.99 (0.96,1.02) | 0.99 (0.95,1.02) | 0.97 (0.92,1.02) |
| cg15239796 | 1.01 (0.94,1.10) | 0.99 (0.90,1.09) | 0.97 (0.84,1.12) |
| cg16747973 | 0.99 (0.95,1.03) | 0.99 (0.96,1.03) | 0.97 (0.91,1.03) |
| cg27611830 | 0.99 (0.96,1.03) | 0.99 (0.96,1.03) | 0.98 (0.94,1.03) |
| cg00257769 | 1.04 (0.97,1.11) | 1.03 (0.96,1.10) | 1.07 (0.98,1.17) |
| cg08808042 | 1.02 (0.98,1.06) | 1.02 (0.98,1.06) | 1.03 (0.96,1.10) |
| cg09589360 | 0.98 (0.94,1.02) | 0.98 (0.94,1.02) | 0.97 (0.92,1.02) |
| cg11683511 | 1.05 (0.99,1.12) | 1.05 (0.98,1.12) | 1.17 (1.02,1.35) |
| cg12869679 | 1.00 (0.97,1.02) | 1.00 (0.97,1.03) | 0.99 (0.96,1.03) |
| cg13161621 | 1.03 (0.97,1.08) | 1.02 (0.97,1.08) | 1.03 (0.95,1.11) |

**Supplemental Table II:** Hazard ratio and 95% CI associating epigenetic aging with all-cause mortality (1) unadjusted, (2) age, and (3) age, and race.

| **Deaths (*n=12)*** | **Horvath** | | **Hannum** | |
| --- | --- | --- | --- | --- |
| **Age Acceleration** | **HR (95% CI)** | **p-value** | **HR (95% CI)** | **p-value** |
| Unadjusted | 0.95 (0.91, 0.99) | 0.01 | 0.97 (0.92,1.01) | 0.15 |
| Age-adjusted | 0.95 (0.89, 0.99) | 0.03 | 0.98 (0.92,1.04) | 0.4 |
| Age and Race-adjusted | 0.94 (0.90,0.99) | 0.03 | 0.97 (0.91, 1.03) | 0.36 |
